# Endophytic Fungi Co-Culture: An Alternative Source of Antimicrobial Substances

**DOI:** 10.3390/microorganisms12122413

**Published:** 2024-11-25

**Authors:** Lucas Silva Tironi, Lucilene Bento Carletto, Eliane Oliveira Silva, Jan Schripsema, Jaine Honorata Hortolan Luiz

**Affiliations:** 1 Institute of Chemistry, Federal University of Alfenas, Alfenas 37130-001, MG, Brazil; lucas.tironi@sou.unifal-mg.edu.br (L.S.T.); luhkarletto@gmail.com (L.B.C.); 2 Department of Organic Chemistry, Chemistry Institute, Federal University of Bahia, Salvador 40170-115, BA, Brazil; elianeos@ufba.br; 3 Metabolomics Group, Laboratory of Chemical Sciences, Universidade Estadual do Norte Fluminense, Campos dos Goytacazes 28013-602, RJ, Brazil

**Keywords:** endophytic fungi, co-culturing, fermentation, specialized metabolites, antimicrobial activity

## Abstract

Antimicrobial resistance is becoming a critical issue due to the widespread and indiscriminate use of antibiotics and antifungals to treat common infections, leading to a growing shortage of effective drugs. Moreover, the increase in antimicrobial resistance is enhancing the pathogenicity and virulence of various pathogens. Microorganisms are key sources of chemically diverse specialized metabolites, which are produced in the final stages of their growth cycle. These metabolites hold significant value in chemical, pharmaceutical, and agrochemical industries. One of the major challenges researchers face in this field is the frequent isolation of already-known substances when classical protocols are used. To address this, several innovative strategies have been developed. The co-culture approach is a powerful tool for activating silent biosynthetic gene clusters, as it simulates natural microbial environments by creating artificial microbial communities. This method has shown promising results, with new compounds being isolated and the yields of target substances being improved. In this context, this review provides examples of antimicrobial compounds obtained from co-cultures of endophytic fungi, conducted in both liquid and solid media. Additionally, the review discusses the advantages and challenges of the co-culture technique. Significance and Impact of the Study: Microbial co-culture is a valuable strategy for discovering new natural products with antimicrobial activity, as well as for scaling up the production of target substances. This review aims to summarize important examples of endophyte co-cultures and highlights the potential of endophytic fungi co-culture for pharmacological applications.

## 1. Introduction

Since the discovery of the first antimicrobials, pathogen resistance to commercial drugs has posed a significant challenge. The emergence of resistant pathogenic bacteria and fungi presents a difficult problem for healthcare systems worldwide, as pathogens can develop new protective mechanisms to resist available medications. In this context, discovering new, effective antimicrobials is crucial to mitigating this global issue [1,2].

Endophytic fungi are valuable resources for the discovery of new natural products, as they have been widely used for medicinal, agricultural, and industrial purposes [3,4]. They have gained considerable attention in bioactive substance detection programs due to their ability to sustainably produce microbial biomass through cultivation in industrial bioreactors [5].

The discovery of endophytes capable of producing novel bioactive substances, or even the same substances as their host plants, has generated high expectations for large-scale compound production via fermentation processes to meet market demands and human needs. However, challenges remain, such as low metabolite production during fermentation and reduced substance production due to the lack of communication between the fungus and its host. When the fungus is not in its natural habitat, gene silencing can occur in axenic monocultures, complicating the production process [6].

To investigate the production of specialized metabolites and activate silent biosynthetic genes for obtaining target compounds, numerous strategies have been developed. One such strategy is co-culture, an approach that induces interactions between microorganisms, triggering the biosynthesis of new natural products or their production on a larger scale, even when the biosynthetic pathways are unknown [7]. The discovery of the antibiotic penicillin by Alexander Fleming in the early 20th century, produced from a co-culture of *Penicillium notatum* and *Staphylococcus aureus* in the same environment, remains one of the most successful examples of this approach [8].

Recently, significant progress has been made in research on co-culture methodologies, particularly those involving two or more species of microorganisms in a shared environment.

This review highlights the effectiveness of microbial co-culture and underscores the untapped potential of endophyte co-cultures in producing compounds with antimicrobial activities, an area that has been relatively unexplored.

## 2. Endophytes and Their Host Plant Interactions

Endophytes are microbes, such as fungi and bacteria, that live within plant tissues without causing any apparent symptoms to their hosts. They have been isolated from a wide variety of plants across many species. Endophytes can inhabit both aboveground and belowground plant parts, making the leaves, stems, and roots of healthy plants potential niches for isolation. In general, woody plants harbor a broader range of endophytes compared to grasses, which results in less host specificity among endophytes [9].

Plant endophytes and symbionts are promising sources from which novel bioactive compounds can be isolated. Recent studies suggest that some important compounds, initially thought to be produced by plants, may actually result from interactions between plants and microbes or between microorganisms themselves. Bioactive specialized metabolites play a crucial role in these interactions within microbial communities, and they may be involved in mechanisms such as parasitism, competition for space or nutrients, or the induction of plant defense responses [10].

Endophytes interact with host plants, and their abundance and diversity vary due to factors such as geographic location, host age, and plant species [11]. They can engage in harmonious interspecific interactions with their hosts in two primary modes: mutualism, in which both the fungus and plant benefit from a close association, or commensalism, in which only one species benefits without harming the other [12]. However, these relationships can change, as fungi are susceptible to alterations caused by biotic factors (e.g., insects, herbivores, parasitic nematodes, and phytopathogenic microorganisms) or abiotic factors (e.g., pH, temperature, water stress, strong winds, salinity) throughout their life cycle [13].

Endophytes can also perform important functions for plants, such as controlling phytopathogens [14], acting as bioremediation agents for xenobiotic compounds [15], promoting plant growth [16], and helping plants adapt to their environment through the biosynthesis of specialized metabolites [17]. As a result, they play a crucial role in enhancing plant resistance to various stresses [18,19].

## 3. Endophytes and Their Metabolites

Natural products discovered in plants and microorganisms have been used by humans for various purposes since ancient times. Microorganisms, including bacteria and fungi, produce many specialized metabolites that can function as bioactive substances or serve as prototypes for developing new products. Due to their chemical diversity and distinct biological activities, the search for microbial substances has been driven by the goal of discovering useful compounds with applications in various fields, such as pharmaceuticals, agrochemicals, and other industrial sectors [1,20].

Among microorganisms, endophytic fungi are particularly notable. These fungi live in association with host plants for all or part of the plant’s life cycle, colonizing healthy plant tissues either intracellularly or intercellularly, without causing any symptoms or apparent diseases [21]. It is important to note that endophytic fungi can synthesize new substances or even those produced by their hosts through symbiotic relationships. They also exhibit excellent potential as biocontrol agents in wastewater treatment and can be cultivated on a large scale [22].

The substances produced by endophytic fungi belong to various chemical classes, including polyketides, shikimic acid derivatives, terpenes, alkaloids, and peptides, among others [5]. However, the literature suggests that many genes in these microorganisms remain inactive when cultivated in synthetic media in axenic cultures, thereby concealing their full biosynthetic potential [23,24].

Since the majority of microorganisms exist as microbial consortia in their natural habitats, microbial co-cultures have been reported to be an efficient way to generate compounds with antifungal and/or antibacterial activity. This occurs through the activation of previously hidden biosynthetic pathways, which are triggered in the presence of other microbes [25].

## 4. The Influence of the Culture Medium in Co-Cultivations

Secondary metabolite production via microorganisms can be significantly affected by environmental factors, which may include nutrient availability, cultivation methods, temperature, incubation time, pH, light, and humidity. Of these various factors, the culture medium’s composition plays a key role in directly influencing the type and quantity of metabolites produced. Variations in nutritional composition can substantially influence the final metabolic profile [26]. Medium composition is even more important in co-culture systems, since metabolite production can vary due to stress from competition among microorganisms within the same environment. This competition is not only for essential nutrients, e.g., salts and minerals, but also for oxygen availability and direct cell-to-cell interactions during fermentation [27].

Although medium composition clearly influences microorganism growth and interactions, few studies have focused on optimizing medium components for co-culture systems [28]. The identification of a medium that effectively supports multiple microorganisms in a co-culture system while also maximizing metabolite productivity merits investigation [29]. During fermentation, the carbon source plays a key role in promoting growth, supporting biomass formation, and providing carbon units for secondary metabolite synthesis [30]. Similarly, the nitrogen source is critical for protein and nucleic acid production, and supplies nitrogen units required for secondary metabolite biosynthesis.

Xu et al. [29] examined how changes in macronutrient composition—e.g., carbon sources, peptides/amino acids, and essential nutrients—affect co-culture media. They suggested that interactions among these macronutrients are crucial for optimizing growth and metabolite productivity. Similarly, Perera et al. [31] found that inorganic nitrogen sources are vital for symbiotic interactions in co-cultures. They observed that altering the types and ratios of these nitrogen sources significantly impacts growth, nutrient uptake, and the production of extracellular metabolites necessary for beneficial interactions between microorganisms.

Design of Experiments (DOE) is a statistical tool commonly used to optimize culture media composition [32]. It identifies variables that influence processes or product responses, providing a statistical foundation for adjusting these variables to enhance performance, reduce costs, and increase the accuracy of results [32]. Of the various DOE methods, the response surface methodology is especially effective for finding optimal conditions for co-cultivation processes [33]. This technique helps identify the best combinations of variables to meet the nutritional needs of microorganisms, reducing unnecessary components in the culture medium, and optimizing the process in fewer experiments. The response surfaces generated when employing this approach are essential for achieving ideal conditions and improving both process efficiency and effectiveness [33].

## 5. Microbial Co-Cultures: Advantages and Challenges

**Figure 1:**
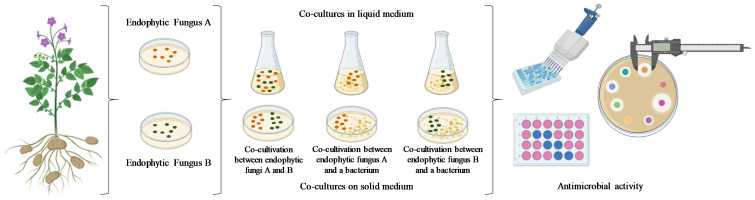
Flowchart for isolation, cultivation in solid and liquid media, and antimicrobial evaluation using fungal co-culture strategy.

The co-culture technique, also known as mixed culture or mixed fermentation, involves cultivating two or more species of microorganisms in either solid or liquid culture media to induce the biosynthesis of specialized metabolites (Figure 1). Co-cultures, in which multiple organisms coexist and grow in contact within a medium, can occur either naturally or artificially. When co-culture is conducted artificially, i.e., in vitro, it allows for the controlled study and development of compounds with high added value. In contrast, natural co-cultures involve mutualistic or commensal relationships, where substances such as phytohormones and growth promoters are produced. However, parasitism or predation can also occur, leading to the release of toxins, antibiotics, antifungals, and alkaloids capable of inhibiting microbial growth [10,34].

An artificial co-culture is designed to simulate the natural ecosystem of the involved organisms, creating a competitive environment in which bioactive substances are produced to ensure the survival of the species [35]. In this type of culture, microorganisms share the same space and nutrients, allowing interactions between species through signaling and/or defense molecules. These interactions can lead to the production of substances of biotechnological or pharmaceutical interest [36]. The presence of different microorganisms in the culture promotes the synthesis of specialized metabolites, as competitive natural environments efficiently stimulate microbial interactions. This strategy has been successfully employed to activate the secondary metabolism of microorganisms, leading to the production of new compounds that are otherwise undetectable in pure cultures [35,36].

Microorganism co-culture is increasingly being recognized as an effective strategy for the large-scale production of pharmaceuticals and nutraceuticals, playing a significant role in the bioremediation and bioenergy sectors. Co-culture systems offer great biotechnological potential due to their versatility and robustness. Artificial co-cultures address the limitations of monocultures by exploiting interactions such as allelopathy. However, further studies are required to gain a deeper understanding of microbial interactions, which could lead to biotechnological advancements and provide more economical methods for generating bioproducts [37].

Microbial interactions rely on both macromolecules and small molecules used for communication, whether within the same species (intraspecies) or between different species (interspecies) [38]. Symbiotic, antagonistic, or allelopathic interactions between microorganisms can involve physical or chemical processes. Among chemical interactions, there may be those involving infochemicals, special signaling molecules (quorum sensing), adhesion factors (biofilms), and secondary metabolites [39]. During these interactions, one microorganism may induce the production of a new substance or activate cryptic biosynthetic pathways in the other. A deeper understanding of these microbial interactions could assist in constructing high-performance consortia, enabling more efficient generation of desirable products [40].

Interpreting microbial interactions is challenging due to the complexity of microbiomes. Since the synergistic interactions between co-cultured microorganisms are species-specific, similar effects cannot be achieved with species from the same genus, highlighting the need for individual evaluation of each interaction [41]. Additionally, microbial communities are highly sensitive to both abiotic and biotic stresses, which can influence the substances produced during the co-culture process [37].

When planning a co-culture, it is essential to consider the objectives, whether for biomass generation, substance production, or even a cleaning system. Once the objective is defined, selecting appropriate microorganisms and optimizing growth parameters for their interaction are crucial to achieving the desired outcome. Therefore, understanding trigger–response mechanisms will pave the way for improving bioprocesses [10].

Co-cultures offer many benefits, such as reduced contamination compared to axenic cultures, shorter cultivation times, induction of new metabolic pathways for the production of novel compounds, increased target product yields, sustainable production of high-value substances, and reduced costs associated with sterilization and feedstock, among others [36]. However, one of the main disadvantages of co-cultures is their limited ability to fully replicate real-world conditions [42].

Liquid cultures provide a convenient and effective way to co-culture different microbial species. In this format, the mixing of species is relatively straightforward, facilitating metabolite exchange and triggering the biosynthesis of new natural products. However, growth interference between co-cultured species can be more pronounced. Therefore, suitable cultivation conditions are essential to ensure compatible coexistence and effective collaboration for the biosynthesis of target products [43,44].

Several techniques are used to conduct co-cultures of microorganisms, each suitable for different microorganisms and specific objectives. The composition of the culture medium plays a crucial role in shaping the interactions between species. The main techniques include the following:

(1) Growth in liquid medium: microorganisms come into direct physical contact;
(2) Solid–liquid interface systems: encapsulation of microorganisms co-cultured in a liquid medium;
(3) Separation by membranes: microorganisms are separated by permeable membranes;
(4) Spatial separations: monocultures are inoculated separately without direct physical contact, but can interact within a shared space;
(5) Microfluidic systems: used primarily in mammalian research, offering better control over fluids and microenvironments [37].

Evaluating each co-culture method allows for the identification of obstacles to overcome in future projects. After selecting a species for co-culture to produce a target substance, parameters such as pH, temperature, agitation, medium composition, and inoculum size must be optimized to achieve high bioproduct yields while ensuring economic viability. Additionally, factors like inoculation rate and the timing of monocultures’ exposure to one another are critical for establishing a balanced co-culture [45,46].

This type of co-culture method is particularly useful for increasing biomass yield in fermentation processes and for producing biofuels, nutraceuticals, and chemicals, where enhancing the growth of the primary microorganism can lead to higher bioproduct yields [47].

In a review by Zhuang and Zhang [7], advances in the use of co-cultures between species based on co-cultivation in liquid and solid media were highlighted, particularly in the biosynthesis of natural products such as polyketides and alkaloids. The authors reported that submerged co-culture facilitated metabolite exchange and the production of new compounds; however, controlling the cultivation conditions was crucial for ensuring the coexistence of the involved species [7].

Many co-culture studies have been conducted without sufficient understanding of the underlying mechanisms. While the use of co-cultures for biosynthesizing new natural products is an established approach, significant progress has been made in recent years. Nevertheless, challenges remain in advancing this promising field of research. The mechanisms behind the biosynthesis of many substances produced through microbial co-culture are still unknown. Additionally, knowledge about cryptic biosynthetic genes and methods to induce gene expression in co-culture remains limited [48].

It is essential to understand and replicate the metabolic synergy between microorganisms in co-culture, stabilize byproducts within this environment, reduce costs associated with separation and purification of the target product, and simulate the natural habitat of microorganisms to determine their compatibility for co-culture [9].

Xu et al. [49], through extensive bibliographic research using the DNP database (Dictionary of Natural Products) and SciFinder, described 158 compounds that were isolated and characterized using the co-culture strategy for fungi in their review. The authors comprehensively summarized various fungal co-culture methods, the substances derived from these co-cultivations, and their biological activities. They concluded that this strategy will continue to play a crucial role in the discovery of novel specialized metabolites and drug development in the future [49].

The co-culture of endophytes is still not extensively explored in terms of increasing metabolite production. Many studies in the literature focus on co-culture as a means of producing new or specific metabolites; however, research aimed at increasing the yield of target molecules in endophyte cultivation (whether bacterial or fungal) is scarce, and this topic requires more attention from researchers.

In this review, co-cultures in liquid and solid media will be discussed, as these methods can simulate real-world conditions in which microorganisms from the same niche are isolated and cultivated together. This approach could lead to more successful co-culture outcomes. Additionally, examples of co-culture between endophytic fungi and other microorganisms, including both pathogenic and non-pathogenic species, that result in the production of compounds with antimicrobial activity will also be highlighted.

## 6. Antimicrobial Substances from Co-Cultivation of Endophytic Fungi

The co-culturing of microorganisms, particularly fungi, has been investigated in recent years as a promising strategy to increase chemical diversity and discover new bioactive compounds. By cultivating different species of fungi in the same environment or combining fungi with bacteria, it is possible to “activate” secondary metabolic pathways that may remain inactive in monocultures. This innovative approach has shown the ability to generate a variety of specialized metabolites, many of which exhibit antimicrobial activity, as evidenced in the current literature [50].

**Figure 2:**
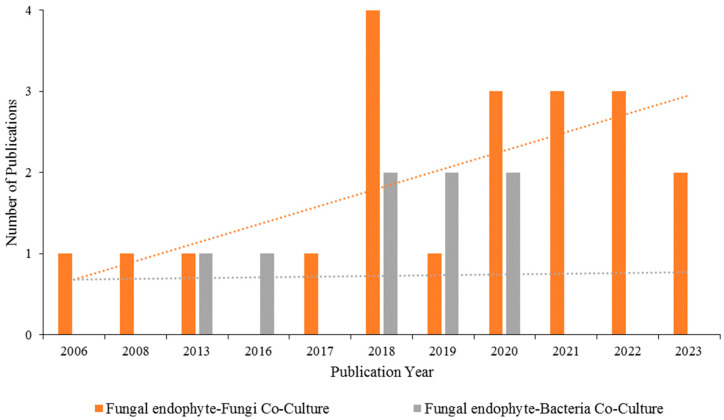
Number of manuscripts published between 2006 and 2023 on endophytic fungi co-culture and the production of antimicrobial compounds (search terms: endophytic fungi, co-culture, and antimicrobial; platforms: Web of Science, SciFinder, Scopus).

Figure 2 shows the number of manuscripts published between 2006 and 2023, reviewed in this study, that focus on the production of antimicrobials in co-cultures of endophytic fungi isolated from healthy plant tissues with other fungi (in orange) and with bacteria (in gray). The trend lines indicate a greater number of publications on fungal endophyte–fungi co-cultures (orange) compared to fungal endophyte–bacteria co-cultures (gray). This could be because fungal–fungal interactions are better understood, whereas interactions between fungi and bacteria are less explored [51].

Although the number of papers on this subject is still limited, significant growth is expected in the coming years due to advances in techniques for producing antimicrobial molecules and the development of new approaches to combining co-cultures in competitive environments [52]. Furthermore, the indiscriminate use of antibiotics and antifungals has led to increased pathogen resistance [53], underscoring the importance of this research.

**Figure 3:**
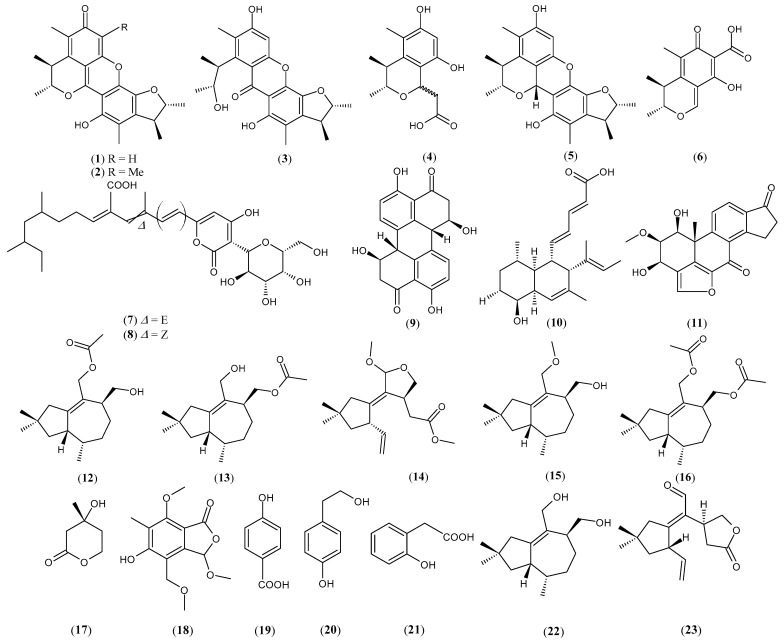

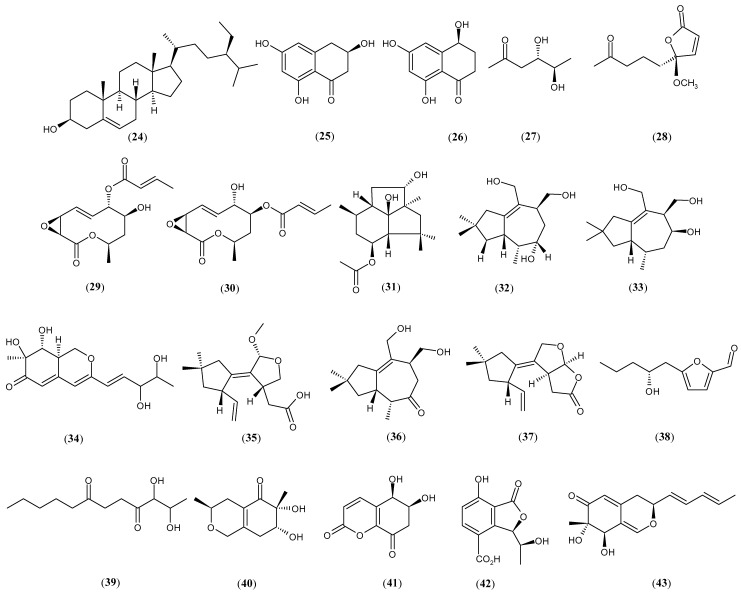

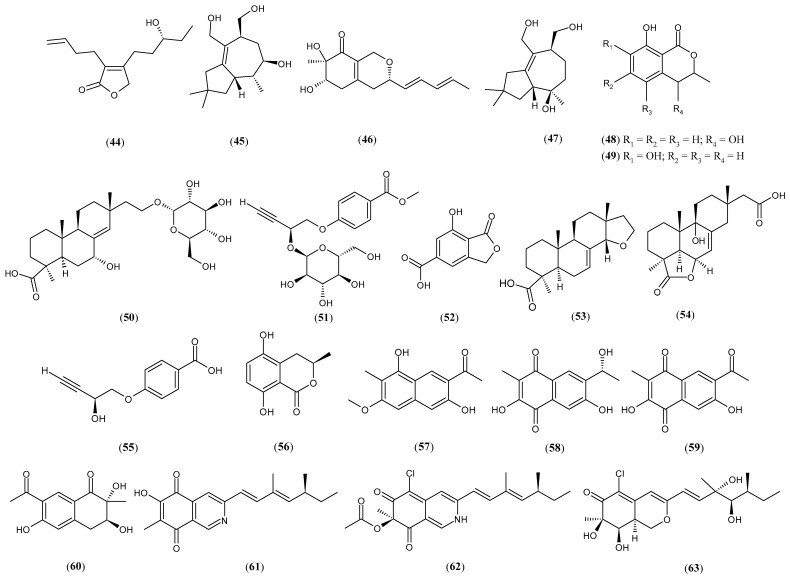

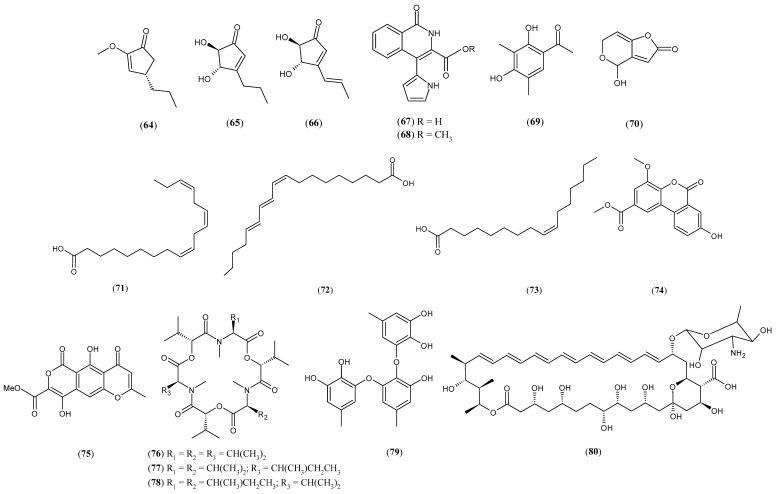

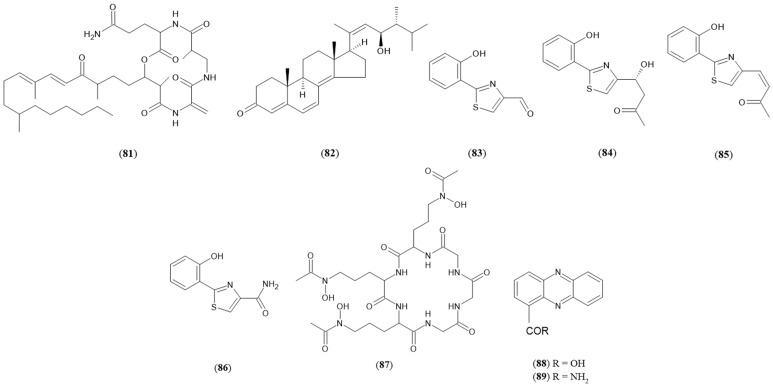
Structures of compounds with antimicrobial activities isolated from endophytic fungi co-culture.

Several antifungal and antibacterial agents have been identified through co-culture experiments using endophytic fungi with other fungi or bacteria in both solid and liquid media. These co-cultures have demonstrated significant potential in the production of antibacterial and antifungal compounds. Some examples of co-cultures are presented in Table 1, which lists the microorganisms involved and their respective metabolites. The chemical structures of these metabolites are illustrated in Figure 3, highlighting their chemical diversity and potential in the discovery and development of new drugs to combat bacterial and fungal infections.

Analyzing Table 1, we can observe some key aspects of the success of co-cultivations in antimicrobial production. There is a noticeable trend in the use of endophytic fungi such as *Irpex lacteus*, *Nigrospora oryzae*, and species of *Penicillium* or *Aspergillus*. Additionally, the use of Gram-positive bacteria, particularly *Bacillus* and *Streptomyces*, stands out. These choices reflect the potential of these microorganisms in bioactive compound synthesis, indicating that such combinations are promising for obtaining effective antimicrobials (Table S2). However, the criteria for strain selection in co-cultures can vary significantly. For researchers focused on discovering new compounds, there is a need for greater clarity on how to translate laboratory-scale co-culture observations into processes that generate relevant quantities of pure and structurally diverse compounds [50].

Some examples from Table 1 are described below. The co-culture of *Aspergillus sydowii* EN-534 and *Penicillium citrinum* EN-535, both endophytes isolated from a marine alga, was performed on a solid medium. The ethyl acetate (EtOAc) extract was purified, yielding 10 citrinin analogs, including a new citrinin dimer (*seco*-penicitrinol A) that showed inhibitory activity against *Vibrio alginolyticus*, with a minimum inhibitory concentration (MIC) of 32.0 μg·mL^−1^, and a new citrinin monomer (penicitrinol L), which demonstrated antibacterial activity against *Escherichia coli*, *V. alginolyticus*, and *Edwardsiella ictaluri*, with an MIC of 64.0 μg·mL^−1^. Among the compounds isolated from this co-culture, some known compounds, such as penicitrinol A, also exhibited antimicrobial activity, with an MIC of 4.0 μg·mL^−1^ against *Micrococcus luteus* [54].

The known compounds D8646-2-6 and iso-D8646-2-6 were isolated from the co-culture of two endophytic fungi, *Camporesia sambuci* FT1061 and *Epicoccum sorghinum* FT1062, both isolated from *Rhodomyrtus tomentosa*. According to the authors, both compounds exhibited weak activity against *Aspergillus niger* and *Paecilomyces lilacinus*, with an MIC of 32.0 μg·mL^−1^ [55].

Chagas et al. [56] observed that the production of stemphyperylenol significantly increased in a culture medium when *Alternaria tenuissima* was grown in co-culture with *Nigrospora sphaerica*, isolated from the *Smallanthus sonchifolius* plant [56]. This finding supports the potential of stemphyperylenol as an antifungal agent, with possible applications in agriculture and in the treatment of *Aspergillus fumigatus* infections [62]. The authors concluded that interactions between endophytes from the same host stimulated the production of natural products capable of controlling fungal growth without harming the host plant [56].

The co-culture of *Hypoxylon fraxineus* and *Hypoxylon rubiginosum* yielded the known phytotoxin viridiol, which was detected as a major metabolite of the ash pathogen in co-culture, as well as the antifungal metabolite phomopsidin, the major secondary metabolite of *H*. *rubiginosum*. The antimicrobial activities of both compounds were evaluated using a serial dilution assay against several bacteria and fungi, revealing an MIC of 66.7 μg·mL^−1^ [57].

The co-cultivation of the endophyte *I. lacteus* with the phytopathogen *N. oryzae* and the entomopathogen *Beauveria bassiana* yielded five new tremulane sesquiterpenoids. All compounds isolated from this co-culture demonstrated significant antifungal activity against several strains. The new compound nigpexin B showed an MIC of 2.0 μg·mL^−1^ against *B*. *bassiana*, comparable to the control nystatin. Additionally, tyrosol, scytalone, 4,6,8-trihydroxy-3,4-dihydronaphthalen-1(2H)-one, and (3*S*,4*R*)-3,4-dihydroxypentanoic acid also exhibited an MIC of 2.0 μg·mL^−1^ against *I. lacteus* or *N. oryzae*. The authors concluded that such a co-culture of phytopathogen–endophyte–entomopathogen could be used for the biological control of insect pests and plant pathogens [58].

On the other hand, the co-culture of the host plant *Dendrobium officinale*, the *I. lacteus* endophyte, and the pathogenic *N. oryzae* yielded nigrolactin, which exhibited antifungal activity against *A. fumigatus*, with an MIC of 1.0 μg·mL^−1^. This suggests that interactions among the host plant, endophyte, and phytopathogen in a co-culture may induce the biosynthesis of novel antifungals [59].

According to Chen et al. [60], co-cultivation between *Nigrospora* sp. and *Stagonosporopsis* sp. endophytes, induced by the host *Nicotiana tabacum* medium, generated an EtOAc extract from which the compounds nigrolactone, multiplolide B, and 4β-acetoxyprobotryane-9β,15α-diol were purified. These compounds showed antifungal activity against *Phomopsis* sp., with MICs of 8.0, 8.0, and 64.0 μg·mL^−1^, respectively [60].

Zhou et al. [61] described the biosynthesis of conocenol B, produced by *I. lacteus* through the induction of *N. oryzae* in a co-cultivation medium. The compound exhibited selective antifungal activity against its co-culture partner *N. oryzae*, with MICs of 16.0 μg·mL^−1^ and 128.0 μg·mL^−1^ against *I. lacteus*. The authors suggested that the fungus can metabolize new compounds to inhibit the growth of the co-cultured fungus while not inhibiting its own growth [61].

According to Wang et al. [63], the co-cultivation of *I. lacteus* and *Armillaria* sp., induced by the host plant *Gastrodia elata*, produced 2,3-dihydroxydodecane-4,7-dione, which demonstrated significant selectivity for antifungal activity against three pathogens but not against *Armillaria* sp. This compound was also beneficial to *G. elata*–*Armillaria* symbiosis. The production of metabolites from *I. lacteus* was inhibited by the co-culture with *G. elata, Armillaria* sp., and *I. lacteus*. 2,3-dihydroxydodecane-4,7-dione was the most abundant in terms of content, accounting for 27.4% and 69.3% of the isolated compounds from the monoculture and the co-culture, respectively [63].

A new microbial culture method, termed “black-box” co-culture, was proposed by Lv et al. [64] for the discovery of new antimicrobial compounds from *Distylium chinense*-associated endophytes. In this method, the biomass of the host plant is added to the culture medium. The compounds chinoketide A, chinoketide B, and xylarphthalide A exhibited antibacterial activity against *Erwinia carotovora*, with MICs of 20.5, 30.4, and 10.2 μg·mL^−1^, respectively [64].

Zhang et al. [65] stated that the metabolic mechanisms in mixed cultures, by favoring the survival of different fungi, can promote the biosynthesis of new chemical structures. In this context, the co-culture between *N. oryzae* and *B. bassiana* produced nigbeauvin A, which exhibited antibacterial activity against *Bacillus subtilis*, with an MIC of 128.0 μg·mL^−1^ [65].

Wu et al. [66] isolated the compounds butenolide irperide, lactedine, and conocenol from the co-culture of the endophyte *I. lacteus* and the pathogenic *N. oryzae*. These compounds exhibited significant antifungal activity against *A. fumigatus*, with MIC of 1.0, 2.0, and 1.0 μg·mL^−1^, respectively, compared to the control nystatin (MIC: 1.0 μg·mL^−1^) [66].

The compounds cis-4-hydroxymellein and 7-hydroxymellein, synthesized from the co-cultivation of *Saccharicola* sp. and *Botryosphaeria parva*, were evaluated against the phytopathogenic fungi *Cladosporium cladosporioides* and *Cladosporium sphaerospermum*. These substances showed antifungal activity, with detection limits of 5.0 to 10.0 μg and 10.0 to 25.0 μg, respectively, comparable to nystatin, used as a control [67].

The metabolites from co-cultures of *Penicillium chrysogenum*–*Nemania primolutea* and *P. chrysogenum*–*A. fumigatus*, both isolated from *Ziziphus jujuba* and supplemented with host plant extract, differed from those produced in monocultures. The antifungal and antifeedant activities of metabolites from these co-cultures were investigated, and the authors demonstrated that the compounds inhibited the pathogenic fungus *Alternaria alternata*, which causes spots and rot in several plants, with MICs ranging from 1.0 to 256.0 μg·mL^−1^ [68].

Co-culturing the mangrove endophytic fungus *Penicillium sclerotiorum* THSH-4 with *P. sclerotiorum* ZJHJJ-18 resulted in the production of nine new azaphilone derivatives. Among these, peniazaphilone A exhibited moderate broad-spectrum antibacterial activity, with an MIC of 12.5 μmol·L^−1^ [69].

Additionally, the compounds asperterrein, dihydroterrein, and terrein exhibited antimicrobial activity against *Alternaria brassicae*, *E. coli*, *Physalospora piricola*, and *S. aureus*, with MICs ranging from 4.0 to 64.0 μg·mL^−1^. These compounds were produced by a mixed culture of *Aspergillus terreus* and *P. lilacinus*, two endophytes isolated from marine red algae. Interestingly, asperterrein and dihydroterrein were not detected in the axenic cultures of either strain [70].

Marine fungal strains (numbers 1924 and 3893) were isolated from a mangrove plant in Hong Kong and co-cultured, yielding a new 1-isoquinolone analog designated as marinamide and its methyl ester. These compounds were not obtained when either strain was cultured individually under the same conditions. Both compounds exhibited antibacterial activities against *E. coli* (bacteriostatic halo diameter: 1.4 cm for marinamide; 2.0 cm for methyl ester), *Pseudomonas pyocyanea* (0.9 cm for marinamide; 1.7 cm for methyl ester), and *S. aureus* (1.0 cm for marinamide; 1.3 cm for methyl ester) at a concentration of 1.0 mg·mL^−1^ [71].

Zhang et al. [72] concluded that during confrontation with *Pythium ultimum*, *Aspergillus clavatonanicus* inhibited the growth of *P. ultimum*, resulting in the formation of clavatol and patulin as the only bioactive compounds, though with distinct kinetics [72].

In another study, a co-culture of two endophytes, *A. fumigatus D* and *Fusarium oxysporum* R1, isolated from the traditional medicinal plants *Edgeworthia chrysantha* Lindl. and *Rumex madaio* Makino, led to the isolation of α-linolenic acid, α-elaeostearic acid, and palmitoleic acid. These compounds demonstrated efficacy against the human pathogen *S. aureus*, with MICs of 50.0, 100.0, and 25.0 μM, respectively [73].

The co-cultivation of the fungal endophyte *Chaetomium* sp. with the bacterium *B. subtilis* on a solid rice medium resulted in up to an 8.3-fold increase in the accumulation of constitutive metabolites. Additionally, the natural product serkydayn was identified exclusively in the co-cultures, not being detected in axenic fungal cultures. Serkydayn exhibited antibacterial activity against *B. subtilis*, with an MIC of 53 μM [74].

The production of enniatins B1 and A1 increased significantly by 36.3% and 78.0%, respectively, when *Fusarium tricinctum* was co-cultivated with the bacterium *B. subtilis* 168 trpC2. The authors concluded that this increase in the production of bioactive fungal compounds, which inhibit bacterial growth with MICs of 16.0 and 8.0 μg·mL^−1^, highlights the potential of co-cultivation experiments and encourages further research, particularly involving pathogenic bacteria. Moreover, these enniatins demonstrated activity against *S*. *aureus*, *Streptococcus pneumoniae*, and *Enterococcus faecalis*, with MICs ranging from 2.0 to 8.0 μg·mL^−1^ [75].

The metabolite sydowiol B, isolated by HPLC (High-Performance Liquid Chromatography) from the extract of a co-culture of *Aspergillus versicolor* with *B. subtilis*, was never detected or isolated in axenic fungal or bacterial cultures, as demonstrated in the study by Abdelwahab et al. [76]. These findings underscore the potential of fungal–bacterial co-cultivation as a powerful tool for inducing the production of new, often cryptic secondary metabolites. Additionally, sydowiol B exhibited antibacterial activity against *S. aureus*, with an MIC of 19.2 μg·mL^−1^ [76].

Chagas and Pupo [77] concluded that endophytic microorganisms chemically interact and regulate each other’s growth through various competitive mechanisms when sharing the same environment. In their investigation of the interactions between *Phomopsis* sp. FLe6 and *Streptomyces albospinus* RLe7, they observed distinct competitive phenotypes: the slow-growing actinobacterium *S. albospinus* RLe7 demonstrated strong competitive potential, primarily due to the production of antifungal compounds such as amphotericin B, while the fast-growing fungus *Phomopsis* sp. FLe6 was inhibited, likely due to competition for nutritional resources [77].

During the co-cultivation of *F. tricinctum* and *Streptomyces lividans*, compounds with known antibiotic activity were identified, including lateropyrone, enniatins B, B1, and A1, as well as the lipopeptide fusaristatin A. The authors suggest that the production of these metabolites may represent a chemical defense strategy employed by the fungus in response to the competitive environment [78].

A new ergosterol derivative, named 23*R*-hydroxy-(20*Z*,24*R*)-ergosta-4,6,8(14),20(22)-tetraen-3-one, was isolated from the co-cultivation of the endophytic fungus *Pleosporales* sp. F46 and the endophytic bacterium *Bacillus wiedmannii* Com1, both derived from the medicinal plant *Mahonia fortunei*. Wang et al. [79] concluded that the results not only demonstrate that the compound is a potent antibacterial candidate, with an MIC of 100 μg·mL^−1^ and an inhibition halo of 0.71 cm against *S. aureus*, but also highlight the potential of co-cultivation to induce the production of cryptic natural products from endophytes of the same host plant [79].

Bathini et al. [80] reported a rare example of the induction of a silent/cryptic bacterial biosynthetic pathway by the co-culture of the Durum wheat plant root-associated bacterium *Pantoea agglomerans* and the date palm leaf-derived fungus *P. citrinum*. In an in vitro antimicrobial evaluation of the isolated compounds, pulicatin H exhibited the most potent antifungal activity against *P. citrinum*, followed by aeruginaldehyde, pulicatin F, and pulicatin I, with MICs of 25.0 μM, 43.0 μM, 53.0 μM, and 127.0 μM, respectively. These results explain the initial suppression of *P. citrinum* growth in the co-cultivation environment [80].

The study by Moussa et al. [81] explores how fungi can activate silent genetic clusters in *Pseudomonas aeruginosa*, leading to the activation of the bacterial defense system. This activation resulted in the production of various antifungal molecules that inhibited the growth of the endophytic fungus *F. tricinctum*, including enniatins B, B1, and A1, fusaristatin A, phenazine-1-carboxylic acid, and phenazine-1-carboxamide, along with compounds related to quorum sensing mechanisms. There are few reports in the literature on the induction of bacterial secondary metabolites in co-culture experiments, making this study a significant contribution to an understanding of these interactions [81].

According to the literature, there is evidence that co-culture leads to the discovery of new chemicals, not only with antimicrobial activity but also with various other applications. Efforts should be directed toward research involving strategies that facilitate the discovery of new molecules capable of addressing challenges in diverse fields, such as medicine, pharmaceuticals, agriculture, and more.

## 7. Conclusions

In recent years, there has been an increase in the resistance of microbial pathogens to available antimicrobials, highlighting the urgent need for the discovery and development of new, effective antimicrobial agents. To address this problem, scientists are actively seeking new sources of antibiotics, antifungals, and other bioactive compounds capable of combating pathogenic infections and overcoming antimicrobial resistance. Despite the challenges, microbial co-culture between species offers a promising approach to generating new natural products, which will continue to play an important role in natural product research for developing new drugs and agricultural products, as well as in meeting the diverse needs of industrial sectors.

Several studies on mixed cultures of microorganisms (bacteria and fungi) have been reported, many of which involve genetically modified microorganisms aimed at increasing the production of a target substance. Our analysis of studies published between 2006 and 2023 on the co-cultivation of endophytic fungi for antimicrobial production revealed that species such as *I. lacteus* and *N. oryzae*, as well as various *Penicillium* and *Aspergillus* species, were widely investigated, possibly due to their adaptability to co-cultivation systems. However, many endophytic species remain unexplored, and their biosynthetic potential, particularly in co-cultures with other fungi or bacteria, may reveal novel compounds of scientific interest.

Studies on endophyte co-cultivation show great potential in generating valuable insights in microbiology, biotechnology, chemistry, and ecology. Such research can not only accelerate the discovery of new bioactive metabolites but also facilitate the development of new chemical structures or significantly increase the yields of target molecules—key aspects that should not be overlooked. Many challenges remain, and a deeper understanding of microbial molecular networks is still needed. Communication among microorganisms in a co-culture should be a focus of future research, as understanding these microbial interactions could advance biotechnology by providing more sustainable and cost-effective methods for bioproduct synthesis.

In the future, advances in co-cultivation research could contribute significantly to innovative solutions against antimicrobial resistance and other global challenges, bringing substantial benefits to humanity.

## Figures and Tables

**Table 1:** Antimicrobials produced by the co-culture of different endophytic fungi. A version of the table alphabetically organized according to the fungi is presented in the Supplementary Materials (Table S1).

Co-Cultivation of Endophytic Fungi and Fungi	Antimicrobials	References
*Aspergillus sydowii* and *Penicillium citrinum*	Penicitrinone A (**1**) Penicitrinone F (**2**) *Seco*-penicitrinol A (**3**) Penicitrinol L (**4**) Penicitrinol A (**5**) Citrinin (**6**)	[54]
*Camporesia sambuci* and *Epicoccum sorghinum*	D8646-2-6 (**7**) Iso-D8646-2-6 (**8**)	[55]
*Alternaria tenuissima* and *Nigrospora sphaerica*	Stemphyperylenol (**9**)	[56]
*Hypoxylon rubiginosum* and *Hymenoscyphus fraxineus*	Phomopsidin (**10**) Viridiol (**11**)	[57]
*Irpex lacteus*, *Nigrospora oryzae,* and *Beauveria bassiana*	Nigpexin A (**12**) Nigpexin B (**13**) Nigpexin C (**14**) Nigpexin D (**15**) Nigpexin E (**16**) Mevalonolactone (**17**) Microsphaerophthalide F (**18**) *p*-hydroxybenzoic acid (**19**) Tyrosol (**20**) 2-hydroxyphenylacetic acid (**21**) Tremulenediol A (**22**) 11-aldehyde-5,6-seco-1,6(13)-tremuladien-5,12-olide (**23**) *β*-sitosterol (**24**) Scytalone (**25**) 4,6,8-trihydroxy-3,4-dihydronaphthalen-1(2H)-one (**26**) (3*S*,4*R*)-3,4-dihydroxypentanoic acid (**27**)	[58]
*Irpex lacteus* and *Nigrospora oryzae*	Nigrolactin (**28**)	[59]
*Nigrospora* sp. and *Stagonosporopsis* sp.	Nigrolactone (**29**) Multiplolide B (**30**) 4*β*-acetoxyprobotryane-9*β*,15*α*-diol (**31**)	[60]
*Nigrospora oryzae* and *Irpex lacteus*	Conocenol B (**32**) Nigrosirpexin A (**33**) Nigirpexin D (**34**)	[61]
*Setophoma* sp. and *Penicillium brasilianum*	Stemphyperylenol **(9)**	[62]
*Irpex lacteus* and *Armillaria* sp.	Irpexlactin B (**35**) Conocenol B (**32**) 11,12-dihydroxy-1-tremulen-5-one (**36**) 11,12-epoxy-5,6-secotremula-1,6(13)-dien-5,12-olide (**37**) Irpexlacte B (**38**) 2,3-dihydroxydodacane-4,7-dione (**39**)	[63]
Endophytes from the leaves of the plant *Distylium chinense*	Chinoketide A (**40**) Chinoketide B (**41**) Xylarphthalide A (**42**)	[64]
*Nigrospora oryzae* and *Beauveria bassiana*	Nigbeauvin A (**43**)	[65]
*Irpex lacteus* and *Nigrospora oryzae*	Butenolide irperide (**44**) Lactedine (**45**) Conocenol B (**32**) Nigirpexin C (**46**) Tremulenediol A (**22**) (+)-(3*S*,6*R*,7*R*)-tremulene-6,11,12-triol (**47**)	[66]
*Saccharicola* sp. and *Botryosphaeria parva*	*cis*-4-hydroxymellein (**48**) 7-hydroxymellein (**49**)	[67]
*Penicillium chrysogenum*, *Nemania primolutea,* and *Aspergillus fumigatus*	Nemmolutin A (**50**) Penigenumin (**51**) Penemin (**52**) Xylabisboein B (**53**) Xylarenolide (**54**) 4-(2-hydroxybutynoxy)benzoic acid (**55**) 5-hydroxymellein (**56**) Penicilligenin (**57**) Monasone B (**58**) Monasone A (**59**) Monaspurpurone (**60**)	[68]
*Penicillium sclerotiorum* THSH-4 and *Penicillium sclerotiorum* ZJHJJ-18	Peniazaphilone A (**61**) Scleratioramine (**62**) WB (**63**)	[69]
*Aspergillus terreus* and *Paecilomyces lilacinus*	Asperterrein (**64**) Dihydroterrein (**65**) Terrein (**66**)	[70]
Marine fungal strains 1924 and 3893 isolated from a plant in a mangrove	Marinamide (**67**) Methyl ester (**68**)	[71]
*Aspergillus clavatonanicus* and *Pythium ultimum*	Clavatol (**69**) Patulin (**70**)	[72]
*Aspergillus fumigatus* and *Fusarium oxysporum*	*α*-linolenic acid (**71**) *α*-elaeostearic acid (**72**) Palmitoleic acid (**73**)	[73]
**Co-Cultivation of Endophytic Fungi and Bacteria**	**Antimicrobials**	**References**
*Chaetomium* sp. and *Bacillus subtilis*	Serkydayn (**74**)	[74]
*Fusarium tricinctum* and *Bacillus subtilis*	Lateropyrone (**75**) Enniatin B (**76**) Enniatin B1 (**77**) Enniatin A1 (**78**)	[75]
*Aspergillus versicolor* and *Bacillus subtilis*	Sydowiol B (**79**)	[76]
*Phomopsis* sp. and *Streptomyces albospinus*	Amphotericin B (**80**)	[77]
*Fusarium tricinctum* and *Streptomyces lividans*	Lateropyrone (**75**) Enniatin B (**76**) Enniatin B1 (**77**) Enniatin A1 (**78**) Fusaristatin A (**81**)	[78]
*Pleosporales* sp. and *Bacillus wiedmannii*	23*R*-hydroxy-(20*Z*,24*R*)-ergosta-4,6,8(14),20(22)-tetraen-3-one (**82**)	[79]
*Penicillium citrinum* and *Pantoea aggolomerans*	Aeruginaldehyde (**83**) Pulicatin H (**84**) Pulicatin I (**85**) Pulicatin F (**86**) Desferrichrome (**87**)	[80]
*Fusarium tricinctum* and *Pseudomonas aeruginosa*	Enniatin B (**76**) Enniatin B1 (**77**) Enniatin A1 (**78**) Fusaristatin A (**81**) Phenazine-1-carboxylic acid (**88**) Phenazine-1-carboxamide (**89**)	[81]

## Supplementary Materials

The following supporting information can be downloaded at https://www.mdpi.com/article/10.3390/microorganisms12122413/s1, Table S1: Antimicrobials produced by the co-culture of different endophytic fungi. This table is presented in the alphabetic order of the endophytic fungi. Table S2: Antimicrobial activity of compounds obtained from endophytic fungi co-culture (Table 1) against inhibited microorganisms.
