# Supplementary material for: Endophytic Fungi Co-Culture: An Alternative Source of Antimicrobial Substances
Source: Microorganisms. 2024 Nov 25;12(12):2413. doi: 10.3390/microorganisms12122413 (PMC11677400; doi:10.3390/microorganisms12122413)
Supplement: Supplementary file 1 [file microorganisms-12-02413-s001.zip › Supporting Information Table S1.pdf]

TABLE

Table S1. Antimicrobials produced by the co-culture of different endophytic fungi. This table is presented in the alphabetic order of the endophytic fungi.

Co-cultivation of endophytic fungi and fungi	Antimicrobials	References
Alternaria tenuissima and *Nigrospora sphaerica*	Stemphyperylenol (**9**)	[56]
Aspergillus clavatonanicus and *Pythium ultimum*	Clavatol (**69**)<br>Patulin (**70**)	[72]
A*spergillus fumigatus* and *Fusarium oxysporum*	*α*-linolenic acid (**71**)<br>*α*-elaeostearic acid (**72**)<br>Palmitoleic acid (**73**)	[73]
Aspergillus sydowii and *Penicillium citrinum*	Penicitrinone A (**1**)<br>Penicitrinone F (**2**)<br>*Seco*-penicitrinol A (**3**)<br>Penicitrinol L (**4**)<br>Penicitrinol A (**5**)<br>Citrinin (**6**)	[54]
Aspergillus terreus and *Paecilomyces lilacinus*	Asperterrein (**64**)<br>Dihydroterrein (**65**)<br>Terrein (**66**)	[70]
Camporesia sambuci and *Epicoccum sorghinum*	D8646-2-6 (**7**)<br>Iso-D8646-2-6 (**8**)	[55]
Hypoxylon rubiginosum and *Hymenoscyphus fraxineus*	Phomopsidin (**10**)<br>Viridiol (**11**)	[57]
Irpex lacteus and *Armillari*a sp.	Irpexlactin B (**35**)<br>Conocenol B (**32**)<br>11,12-dihydroxy-1-tremulen-5-one (**36**)<br>11,12-epoxy-5,6-secotremula-1,6(13)-dien-5,12-olide (**37**)<br>Irpexlacte B (**38**)<br>2,3-dihydroxydodacane-4,7-dione (**39**)	[63]
Irpex lacteus, *Nigrospora oryzae* and *Beauveria bassiana*	Nigpexin A (**12**)<br>Nigpexin B (**13**)<br>Nigpexin C (**14**)<br>Nigpexin D (**15**)<br>Nigpexin E (**16**)<br>Mevalonolactone (**17**)<br>Microsphaerophthalide F (**18**)<br>*p*-hydroxybenzoic acid (**19**)<br>Tyrosol (**20**)<br>2-hydroxyphenylacetic acid (**21**)<br>Tremulenediol A (**22**)<br>11-aldehyde-5,6-seco-1,6(13)-tremuladien-5,12-olide (**23**)<br>*β*-sitosterol (**24**)<br>Scytalone (**25**)<br>4,6,8-trihydroxy-3,4-dihydronaphthalen-1(2H)-one (**26**)<br>(3*S*,4*R*)-3,4-dihydroxypentanoic acid (**27**)	[58]
Irpex lacteus and *Nigrospora oryzae*	Nigrolactin (**28**)	[59]
Irpex lacteus and *Nigrospora oryzae*	Butenolide irperide (**44**)<br>Lactedine (**45**)<br>Conocenol B (**32**)<br>Nigirpexin C (**46**)<br>Tremulenediol A (**22**)<br>(+)-(3*S*,6*R*,7*R*)-tremulene-6,11,12-triol (**47**)	[66]

Nigrospora oryzae and *Beauveria bassiana*	Nigbeauvin A (**43**)	[65]
Nigrospora oryzae and *Irpex lacteus*	Conocenol B (**32**)<br>Nigrosirpexin A (**33**)<br>Nigirpexin D (**34**)	[61]
Nigrospora sp. and *Stagonosporopsis* sp.	Nigrolactone (**29**)<br>Multiplolide B (**30**)<br>4*β*-acetoxyprobotryane-9*β*,15*α*-diol (**31**)	[60]
Penicillium chrysogenum, *Nemania primolutea* and *Aspergillus fumigatus*	Nemmolutin A (**50**)<br>Penigenumin (**51**)<br>Penemin (**52**)<br>Xylabisboein B (**53**)<br>Xylarenolide (**54**)<br>4-(2-hydroxybutynoxy)benzoic acid (**55**)<br>5-hydroxymellein (**56**)<br>Penicilligenin (**57**)<br>Monasone B (**58**)<br>Monasone A (**59**)<br>Monaspurpurone (**60**)	[68]
Penicillium sclerotiorum THSH-4 and *Penicillium sclerotiorum* ZJHJJ-18	Peniazaphilone A (**61**)<br>Scleratioramine (**62**)<br>WB (**63**)	[69]
Saccharicola sp. and *Botryosphaeria parva*	*cis*-4-hydroxymellein (**48**)<br>7-hydroxymellein (**49**)	[67]
Setophoma sp. and *Penicillium brasilianum*	Stemphyperylenol (**9**)	[62]
Endophytes from the leaves of the plant *Distylium chinense*	Chinoketide A (**40**)<br>Chinoketide B (**41**)<br>Xylarphthalide A (**42**)	[64]
Marine fungal strains 1924 and 3893 were isolated from a plant in a mangrove	Marinamide (**67**)<br>Methyl ester (**68**)	[71]

**Co-cultivation of endophytic fungi and bacteria**	**Antimicrobials**	**References**
Aspergillus versicolor and *Bacillus subtilis*	Sydowiol B (**79**)	[76]
Chaetomium sp. and *Bacillus subtilis*	Serkydayn (**74**)	[74]
Fusarium tricinctum and *Bacillus subtilis*	Lateropyrone (**75**)<br>Enniatin B (**76**)<br>Enniatin B1 (**77**)<br>Enniatin A1 (**78**)	[75]
Fusarium tricinctum and *Streptomyces lividans*	Lateropyrone (**75**)<br>Enniatin B (**76**)<br>Enniatin B1 (**77**)<br>Enniatin A1 (**78**)<br>Fusaristatin A (**81**)	[78]
Fusarium tricinctum and *Pseudomonas aeruginosa*	Enniatin B (**76**)<br>Enniatin B1 (**77**)<br>Enniatin A1 (**78**)<br>Fusaristatin A (**81**)<br>Phenazine-1-carboxylic acid (**88**)<br>Phenazine-1-carboxamide (**89**)	[81]
Penicillium citrinum and *Pantoea aggolomerans*	Aeruginaldehyde (**83**)<br>Pulicatin H (**84**)<br>Pulicatin I (**85**)<br>Pulicatin F (**86**)<br>Desferrichrome (**87**)	[80]
Phomopsis sp. and *Streptomyces albospinus*	Amphotericin B (**80**)	[77]
Pleosporales sp. and *Bacillus wiedmannii*	23*R*-hydroxy-(20*Z*,24*R*)-ergosta-4,6,8(14),20(22)-tetraen-3-one (**82**)	[79]
